# Promisomics and the Short-Circuiting of Mind

**DOI:** 10.1523/ENEURO.0521-20.2021

**Published:** 2021-03-10

**Authors:** Alex Gomez-Marin

**Affiliations:** Instituto de Neurociencias de Alicante, CSIC-UMH, 03550 San Juan de Alicante, Spain

**Keywords:** cognition, connectomics, levels of analysis, promissory materialism

## Significance Statement

Grand neuroscience projects, such as connectomics, have a recurrent tendency to overpromise and underdeliver. Here I critically assess what is done in contrast with what is claimed about such endeavors, especially when the results are “horizontal” and the conclusions “vertical”, namely, when maps of one level (synaptic connections) are conflated with mappings between levels (neural function, animal behavior, cognitive processes). I argue that to suggest that connectomics will give us the mind of a mouse, a human or even a fly is conceptually flawed. Even if we, neuroscientists, do not take our metaphors literally, we should take them seriously.

## 

Imagine that a group of prominent psychologists wrote a paper about the brain of a mouse disregarding neural matters. Measuring attention or perception at unprecedented resolution, these scientists would promise to solve the mouse’s brain (by decree, it would emerge from “mind stuff”). Published in a top cognitive science journal, “The Brain of a Mouse” would inspire the current and generations to come.

Conversely, and in reality, we have it reverse for neuroscience. Mirroring the spirit of the 1986 classic whose running head read “The Mind of a Worm” (White et al., 1986), a recent commentary (Abbott et al., 2020) discusses the feasibility and benefits of mapping the synaptic connections of an entire rodent brain. Entitled “The Mind of a Mouse,” the piece illustrates some of the strengths of the neural zeitgeist. It inadvertently also reveals some of its paradigmatic weaknesses.

What, if anything, could be “wrong” with attempting to map a mouse brain at the synapse level?

In terms of quantity, the authors do the math and set the case: If the connectome of the humble worm were the equivalent in length to the width of a typical airplane seat (by taking 1000 μm^3^ of brain volume as a 1-cm interval), then mapping the connectome of a mouse would involve covering the distance from Boston to Lisbon. The numbers are indeed daunting. But so is the apparent lack of need for conceptual work on the subject. What is there to say about the qualitative nature of the challenge? Borrowing from the Boston-Lisbon analogy, would knowing how far both cities are reveal anything about the purpose of the trip? One could photograph every inch along the way and still remain ignorant about whether one traveled to Lisbon to attend a wedding or a funeral.

This leads to a kind of scientific *déjà vu*. As molecular biologists claim to have explained the nature of life in molecular terms, so do neuroscientists seek to explain the nature of mind in neural terms. Connectomics borrows the *élan* of genomics and directs it to the brain. A connectome (Hagmann, 2005; Sporns et al., 2005) is a comprehensive map of neural connections in the brain, the nervous system architecture at single-synapse resolution. Regarded as the logical (but not biological) analog of an electronic circuit diagram, the quest is not without major caveats (Lazebnik, 2002; Jonas and Kording, 2017). Contrary to what some might still think, genes alone do not determine the development of biological form (arguably one of the two fundamentally unsolved problems in biology, which the latter Watson thought DNA could solve), nor does neural activity by itself give rise to behavior, cognition, mind or the c-word (arguably the other fundamentally unsolved problem, which the latter Crick sought to crack).

Nonetheless, “omics” missions are pellucid: sequence the genome, map the connectome. A theoretical vacuum is supplemented with a methodological *tour de force*, whereby new technology plus big datasets are expected to turn low-level descriptions into high-level explanations. This is combined with the misguided suggestion that the only way to answer big questions is via “big science” (occasionally providing big insights), with its tripartite structure of big money, big tools, big data.

Thus, even if we could, it is unclear whether we should have such a connectome. Before answering how, it may be wise asking what for.

Let us step back. What did we really learn from the worm’s connectome, especially compared with what was promised (and spent)? What did the connectome actually explain? Some may claim that we learned a lot, others that we learned very little. This actually opens a can of worms. If the goal ever was to understand the nematode’s behavior (not to mention its mind, which entails an extra conceptual pirouette), we certainly learned virtually nothing. It is argued that the worm turned out to be “surprisingly” more complex than the funded grantees thought. And yet, to claim that what we learned is that we could not learn much sounds rather cynical.

Despite their particularities, a similar story can be told for the history of grand initiatives such as the Human Genome Project (Hall, 2010; Science, 2001) or the Human Brain Project (Theil, 2015). We have been here before. What the advocates of the genome assured (and failed to deliver) for life, some proponents of the connectome vaticinate for mind: revolutionary understanding and medical miracles. Apparently, “similar objections were voiced when the Human Genome Project, *now widely viewed as a success*, was first proposed” (Lichtman and Sanes, 2008; my italics). As it turns out, hard sells are bought more easily (Bennet, 2013). Politely said, “faced with the need to convince skeptical funders, they needed to present an optimistic view” (Lichtman and Sanes, 2008). In a word, overpromise and underdeliver.

We have had the complete connectome (and genome) of this fantastic humble nematode for many years now. No doubt, such information has helped neuroscientists when designing and interpreting their experiments. But, what does it tell us, precisely, about “the mind of the worm”? Arguably zilch. How come? Because there are too many gaps to bridge, too many levels to connect, too many covert assumptions to spell out.

Synapses are obviously not all you need to characterize a circuit. One needs to know the nature of the connections, as well as cell types. A connectome does not map neural signals either. And, despite the phrase “functional connectivity,” one does not get circuit function from circuit structure, nor causality (Mehler and Kording, 2018). Neurobiology is profuse with instances where the same circuit gives rise to different functions, and where the same function is accomplished by different circuits (Gunaratne et al., 2017). One-to-one mappings are elusive. Moreover, neuromodulation, the biochemical regulation of neural excitability, is pervasive and crucial (Marder, 2012). In addition, the behavior of organisms is not the behavior of their brains. Central nervous systems inhabit species-specific bodies (Chiel and Beer, 1997) that enact a world (Thompson and Cosmelli, 2011). And such environments, rather than being mere physical surroundings, are idiosyncratically meaningful to each organism (von Uexküll, 1909). Brains are overrated (Ghazanfar, 2018). Furthermore, there is variability within (Lu et al., 2009) and across (Witvliet et al., 2020) individual connectomes. Experience constantly reshapes neural circuits. Development matters. Animals are embodied historical beings in a process of extensive becoming (Gomez-Marin and Ghazanfar, 2019).

All these dynamical aspects are nowhere to be found in those maps. Again, structure does matter [even to Marrian functionalists (Marr, 1982), since substrate-independence is not substrate-irrelevance], but the connectomics canon misses so many connections. A few neurons in the lobster’s stomach (or, basically, wherever one looks) attest such difficulties too (Marder and Bucher, 2007). Biology needs technology, but biological unknowns are not an engineering problem. Nor is neuroscience primarily a data science problem either, despite the daunting task of collecting, processing and making sense of such large volumes (Lichtman et al., 2014). This brings us to another set of challenges. How are we, humans, going to make sense of such data? Hopefully, we are not going to leave its interpretation also to machines. One should be prudent not to add to the hypes and hopes of omics those of AI. Wondrous maps may be comprehensive at the expense of being comprehensible. The difference between complicated and complex not of degree but of kind.

And yet, this is already-well-known and dully acknowledged. Deeming the above observations a collection of strawman arguments is somewhat a strawman argument itself since, even when acknowledged *de jure*, such biological points are not granted *de facto*. In other words, research is planned, executed and interpreted without having them into account, until one asks about their importance. And so, extrapolating from the wording of the worm literature, we step into a disappointing infinite regress. We now face the same but also a different challenge: forget the nematode, the time seems to be ripe to map a mammalian brain.

No doubt, this adventure demands fascinating new tools, the usual spur to keep the enterprise running. The collaborative aspect is essential and refreshing. Plus, the more species studied, the better. Not only does neuroscience need behavior (Krakauer et al., 2017; Niv, 2020) but also comparative evolutionary accounts (Laurent, 2016; Cisek, 2019). So far so good. And yet, the rationale of the undertaking is sustained by grand vows articulated with a rhetoric surfeited with “filler verbs” (underly, determine, unravel) and “killer adjectives” (transformative, profound, disruptive, mechanistic, revolutionary). Promethean neuroscience becomes an instantiation of the Sisyphean myth (Hadot, 2006).

Do we need to get the connectome before we realize it cannot bridge the other levels of organization?

Let us be clear, there is *a priori* nothing wrong with industriously performing detailed biological work. Detailed neural mappings in organisms such as the larval fruit fly (Eichler et al., 2017), adult fruit fly (Zheng et al., 2018; Hulse et al., 2020; Li et al., 2020; Scheffer et al., 2020), larval zebrafish (Hildebrand et al., 2017), or mice (Oh et al.,2014; Winnubst et al., 2019; Coletta et al., 2020) shed light onto facts and principles of neural organization. Connectomes can indeed be very useful to understand how simple motor patterns are generated. This is not a black or white issue, but one of subtle grays. One should not subscribe to the all-easy and arrogant critique of biology as mere stamp-collecting. Neither blindly endorse the collection of data for its own sake. The sleight of mind takes place when maps at one level (i.e., synaptic connections) are conflated with mappings between levels (neural function, animal behavior, cognitive processes), namely, when the results are “horizontal” but the conclusions “vertical.”

How is a wiring diagram equated with an organism’s mind?!

Both science and science fiction tend to localize individuality in the brain. In other words, the brain would be “the somatic limit of the self” (Vidal, 2016). Upon transplantation, it is thought that one’s brain would simply miss (like a lover) one’s body, while one would remain essentially the same. And yet, the difference between having and being a brain is crucial (Gabriel, 2017). If the connectome makes me who I am (Seung, 2012), then skipping morning coffee makes me who I am not. The mind is not connecting the dots.

When pressed, the forerunners of the connectome (and of “promisomics,” for that matter) would remark that one should not take certain figures of speech literally. Perhaps not seriously either? The proclaimers of the “Map’ Em All” mantra concede that a future connectome would inform and constrain future work. This is either a splendid truism (the past will somewhat influence the future) or unfathomable futurology (one will have to wait for the future to know), or perhaps both, as a scientific version of the “oracular illusion” (Rosset, 2012). It is a no-brainer that, with or without a question in mind, present research shall enable future research, either as its main goal or as an unexpected consequence of other goals. The insistence that brain function is determined (in part) by how neurons are connected is conceded (notwithstanding logical fallacies, such as those concealed in “necessity and sufficiency” claims; Gomez-Marin, 2017; Yoshihara and Yoshihara, 2018). But this is also true for hormones, microbiota, muscles, or the environment. They all influence brain activity. Do we also need the metabolome, the “ethome,” the “bodyome,” and even the “worldome”? Why? Why not? What would we do with them? An infinite resolution map is worse than the territory.

At the risk of oversimplifying the argument of those determined to obtain the connectome (or making it on their behalf), perhaps the core rationale is this: since we do not know what details matter, all details matter. Omics is the limit case of callow reductionism when the components table tends to infinity. “Without knowing the parts list and what is connected to what, how could one ever really know how it works?” (Morgan and Lichtman, 2013). Should we then collect everything we can, just in case? Can we know what is inessential only later? It is indeed futile and even foolish to argue against more (and better) data in principle, but what about in practice? Producing tons of high-quality “bricks” does not necessarily confer high-quality “buildings” (Forscher, 1963). Can we do without hypotheses? And without theory? For instance, in contrast with the idea that to fully understand one must measure fully, one finds in the notion of coarse-graining insightful overtones for downward causation (Flack, 2017) or causal emergence (Hoel et al., 2013). In other words, coarse-grained objects are not mere lossy summaries of fundamental “building blocks” (once conceived as the ultimate bottom of nature but which dissolve at the ultramicroscale, as physicists discovered more than a century ago, but whose premodern attitude biologists still seek to imitate). The belief that what one sees under a microscope is realer than what we see with bare eyes is an ingrained bias of thought (Capek, 1971). Theoretical work is the art of discarding information, to make informed decisions about what is signal and what is noise. In fact, omitting certain details is not only possible but desirable. Leaving technological challenges and sociological matters aside, the key theoretical question is whether we must collect each and every detail, and why.

Never mind. Unexpected results and fascinating discoveries along the way will help us get through the unfulfilled promises. And, anyhow, if we do not map it, somebody else will. In a kind of perfectly sealed reasoning, it seems that the only way to know whether we need the connectome is to actually have it. The main argument is in fact an assertion: the connectome is mandatory if we stand any chance of understanding the function of nervous systems, the behavior of organisms, and animal minds. Rather than asking why, pioneers prefer to ask why not, articulating and subsequently countering some of the main arguments against connectomics (Morgan and Lichtman, 2013). Where is the burden of proof? Physics pioneered and thrived with “big science” approaches indeed, but it never occurred to a sensible cosmologist to measure the position and momentum of all the atoms in the galaxy, nor to meteorologists to track the trajectories of each and every water droplet falling from a cloud. Again, any map is in principle useful, but how much detail is too much detail?

The first sentence of the abstract of the aforementioned piece (Abbott et al., 2020) is worth pondering: “Large scientific projects in genomics and astronomy are influential not because they answer any single question but because they enable investigation of continuously arising new questions from the same data-rich sources.” But note that any project, and large ones in particular, given the limited nature of scientific funding, attention, and talent, preclude the realization of other projects. Moreover, and borrowing the concept from social media, “audience capture” can easily turn against oneself: writing what you think grant agencies want to read, rather than what you want to do, reinforces a vicious intellectual cycle. Leaving these inconvenient remarks aside, such kind of hypothesis-enabling research should, at least, refrain from grand vague pledges. The last sentence of the paper hammers it: “we predict that a whole-brain mammalian connectome will generate entirely new and unanticipated questions about the nervous system and perhaps represent a turning point in the pursuit of understanding what makes us the unique animals we are.” Well, yes, or perhaps not at all. Who knows? That time will tell is not an argument. To issue promissory notes on top of promissory notes is rather immoderate.

Let us go back to the Human Genome Project. A decade after its launch in 1990, President Bill Clinton proclaimed: “Today we are learning the language in which God created life” (Collins, 2006). If nature is a book, physicists use to read it mathematical language. In turn, molecular biologists pretend to decipher the book of life as if written in genetic code (Kay, 2000). The book of mind would reveal its secrets to neuroscientists in neural jargon. And yet, even when having all the letters, we often remain lost in translation. Text and context are two different things. To Clinton’s statement, editors of eminent journals added: “We will have extra limbs, if we want them, and maybe even wings to fly” (Nature, 2011). Forecasts tell you no less about the forecaster than about the future. Of course, as always, the best is yet to come… The allure and grandeur of “big biology,” the quests for Holy Grails and Rosetta Stones are crowded with implausible pledges. The declaration of what one will do is conflated with the assurance of what will happen. The so-called discovery-driven research, confidently not hypothesis-driven, is often chiefly technology-driven. When academia plays business, when creative scientific discovery adopts the modus operandi of neoliberalism, the resulting creature becomes rather Frankensteinian (Lazebnik, 2018).

To recap, my position should not be taken as a plea against scientific pluralism. Quite the contrary. Nor do I pretend to tell others what to do. I am challenging what is claimed or suggested, especially when the gap between the data and its interpretation, between what is promised and what is delivered, is as great as unbridged. Any approach has limitations. But neuroscientists (of course, far from being considered a homogeneous group) have better work to do than explaining the mind away. Nowadays scientific writing is suffused with “promissory materialism” (Popper and Eccles, 1977). There is a whiff of reductive physicalism: the mind will be automatically understood once we understand the brain (one never really hears how), keep on mapping and it will eventually get cognitive (but even water is not just H_2_O; Chang, 2012). The hope that meaning will emerge from the neural jungle, both for the mouse and for the neuroscientists studying it, is simply a naive philosophical commitment presented as an ensuing scientific fact. What would every single neural connection tell us about a broken heart or a Buddhist monk? Neuro-exceptionalism for mental matters is a myth, in the realm of religion (Thompson, 2020) as well as within science itself. The connectome is certainly not sufficient and, in many ways, probably not even necessary as an explainer of cognition (Barack and Krakauer, 2021). Not an epistemic foundation and even less a replacement for mind. Neural connections will not give us mice minds in the same way that quarks do not explain nor produce stock markets. Optimism and audacity are welcome, but conceit ultimately gives rise to concern. Climbing a tree (no matter how tall) is not the first step to get to the moon (Dreyfus, 2012).

## Synthesis

Reviewing Editor: Erica Glasper, University of Maryland Decisions are customarily a result of the Reviewing Editor and the peer reviewers coming together and discussing their recommendations until a consensus is reached. When revisions are invited, a fact-based synthesis statement explaining their decision and outlining what is needed to prepare a revision will be listed below. The following reviewer(s) agreed to reveal their identity: Federico Adolfi.

Manuscript Title: Nevermind

Manuscript #: eN-COM-0521-20

Synthesis

Within this commentary lies a detailed assessment of the value of connectome mapping strategies within the field of neuroscience. This article was reviewed by two respected leaders in the field of neuroscience and each concluded that the article was well-written, thought -provoking, and shed a light on the many challenges that surround performing these types of investigations, as well as pinpointed some limitations in our ability to draw conclusions from these investigations. The reviewers note that this article will help the larger neuroscience community avoid many epistemic pitfalls due to the authors important and necessary pushback against implicit, reductive assumptions in neuroscience-adjacent fields. Despite this overall enthusiasm, both reviewers agreed that some statements were " overly subjective in nature and not necessary to support the author’s main arguments." The author is encouraged to revise or remove the statements (these statements can be found in italics below.

1) Subjective Statements:

a) "But too soon we come across the usual "promissory notes" using the usual rhetoric based on "filler verbs" (underly, determine, unravel) and "killer adjectives" (transformative, disruptive, mechanistic)."

i) This sentence highlights verbs and adjectives that are often found in scientific literature. The piece suggests that the use of these terms leads to unobtainable goals. However, in many instances, research has been shown to find factors that underlie or determine a certain behavior, or provide a mechanistic understanding. Thus, the negation of these terms draws away from the point that the author is trying to make in the paper.

b) "Who knows? To issue promissory notes on top of promissory notes is a bit too much."

i) This statement is very subjective and not necessary - e.g., what qualifies as being ‘too much’?

c) Line 95: The contention that examining synaptic connections is done by ‘uneducated’ based on an ‘act of faith’ is not warranted. Thus, this sentence should be revised accordingly with removal of these statements.

d) Some statements could be interpreted as value judgements and should be rephrased and substantiated. The risk of leaving the following statements as is could be viewed as antagonizing and polarizing:

i) "This is...unsophisticated futurology."

ii) "The first sentence...is delicious"

iii) "the (uneducated) guess..."

2) The lack of citations and a reference list undermines the weight and value of the arguments. A reader who is not already familiar with the relevant literature may miss the connections that are being made. Citations should be added throughout the article to assist the reader with connecting this well-written critique to the relevant literature. This will help to make the manuscript more educational. This is of particular importance for researchers in computational cognitive neuroscience with backgrounds that are more tech-heavy that lack the background knowledge against a bottom-up, big data approach that is often standard in other areas of neuroscience. Several criticisms, in the absence of support by referencing and quoting, can easily be misinterpreted as attacking a straw-man claim that nobody holds. For example,

a) "one does not get circuit function from circuit structure".

i) It would be hard to get somebody to agree with the extreme opposite, so there is no need to contradict such a hypothetical statement. Instead, one can think of particular cases where the opposite statement holds - where indeed structure has hinted at function - and formulate a critique of that more nuanced view which propels some scientists’ reductionist ideas in practice.

b) One reviewer suggests the "critique needs to point to passages in the original commentary or other parts of the literature where a relative consensus around a false or fallible claim lies. If it cannot be shown directly with quotations, which is perfectly possible in the case of implicit assumptions, then it needs to be argued by, for example, pointing to scientists’ actions stemming from flawed assumptions"

3) The author is encouraged to spell out counterarguments to claims such as this from the target commentary:

a) "These data are generating many hypotheses that are already being put to the test with new experiments. In areas of the fly brain that have seen particularly intense connectomic study, such as the mushroom body, central complex, and visual system, it is increasingly difficult to envision studies that ignore the insights generated by studying their connectomes."

4) Early career researcher may find of particular interest the identification of misplaced assumptions in the section "A path toward designing non-biological thinking systems". Given the hype surrounding AI research, this field if particularly vulnerable in terms of harboring flawed implicit assumptions about how we gain knowledge of how intelligent systems work. The author is encouraged to address the following questions:

a) Are there any mischaracterizations behind the view that "Neuroscience has thus already had a profound impact on the development of AI."?

b) Has this occurred in a bottom-up way as many proponents like to portray it?

c) How exactly is the full connectome a "blueprint for cognitive computing"?

i) It seems this claim in the section mentioned above is particularly weak and unsubstantiated in the target commentary.

5) The author is encouraged to change the manuscript title. The title, as it stands, may turn away readers due to its dismissiveness. The purpose of this commentary is to engage the audience and to convince them to consider your point of view. The title alone could lead our audience to ignore the article altogether.

6) A typo was found here: "in the pursuit of understand"
